# Supplementary material for: Epigenetic Deregulation of Protein Tyrosine Kinase 6 Promotes Carcinogenesis of Oral Squamous Cell Carcinoma
Source: Int J Mol Sci. 2022 Apr 19;23(9):4495. doi: 10.3390/ijms23094495 (PMC9104624; doi:10.3390/ijms23094495)

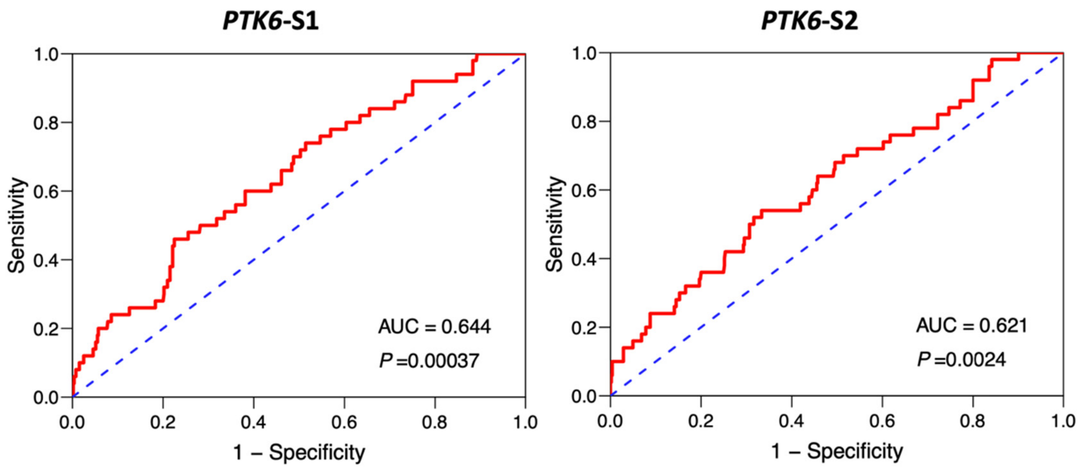

Supplementary Figure S1. The TCGA data were used to show the diagnostic value of *PTK6* hypomethylation in HNSC by plotting the ROC curve.

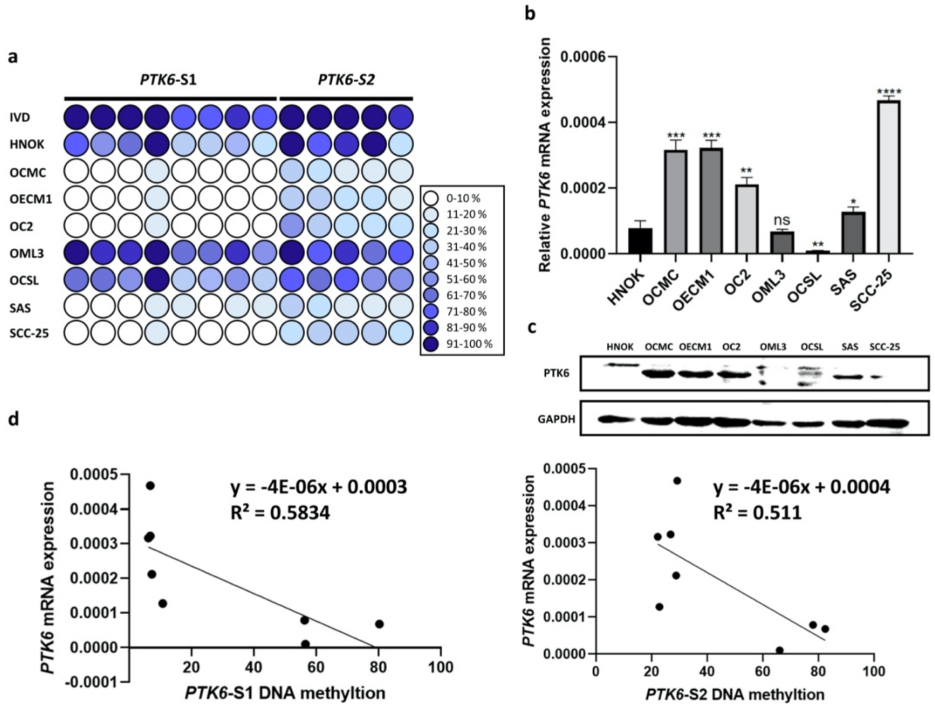

Supplementary Figure S2. Methylation, transcriptional, and translational levels of PTK6 in HNOK and OSCC cell lines. (a) The methylation of *PTK6* was detected in HNOK and OSCC cell lines. (b) The mRNA expression of *PTK6* in HNOK and OSCC cell lines. (c) The protein expression of PTK6 in HNOK and OSCC cell lines. (d) The correction of the methylation of *PTK6* and the *PTK6* transcription level in OSCC cell lines.

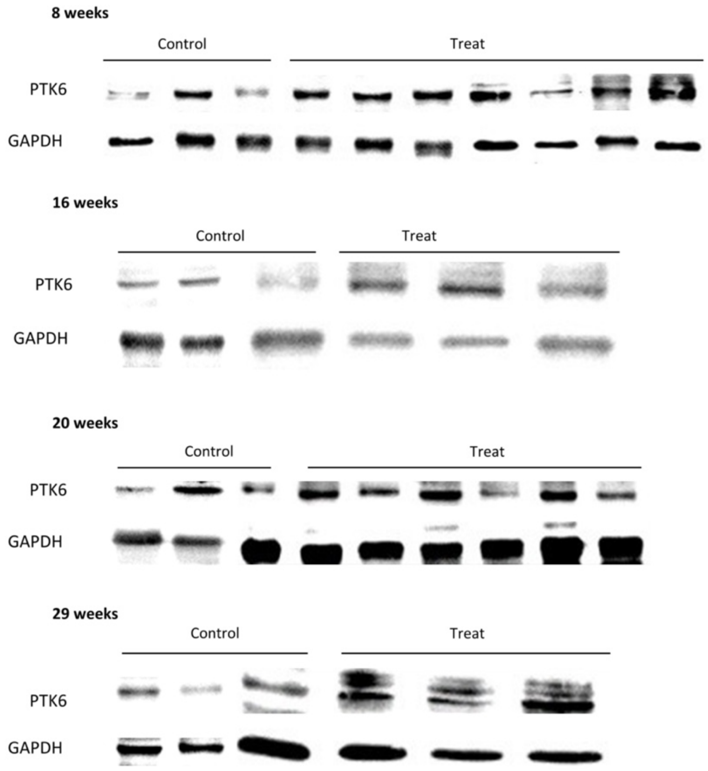

Supplementary Figure S3. Western blot showed the protein expression of PTK6 in the tongue tissues of the OSCC mice at different time points.

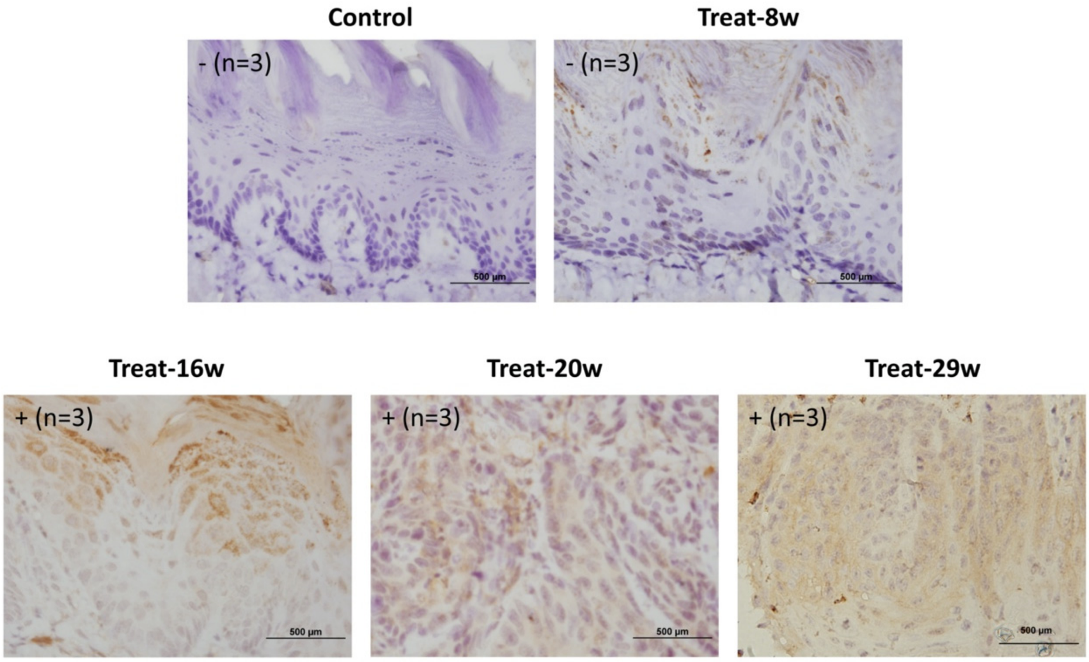

Supplementary Figure S4. Immunohistochemistry staining showed the protein expression of PTK6 in the tongue tissues of the OSCC mice at different time points.

Table S1. The primer sequences were used in bisulfite genomic DNA sequencing and qPCR

**Bisulfite genomic DNA pyrosequencing primer**		
Human-PTK6-F1	**Accession number :** NC_000020.11	GGGATTAGGTTTATTTGGGTTTTAAGTAT
Human-PTK6-R1		CCACTACTCCTCCTTCCTAACCA
Human-PTK6-S1		GGTTTTTGGGATTTTAAGTT
Human-PTK6-F2		GTGGTTAGGAAGGAGGAGTAG
Human-PTK6-R2		CCTAAACAACCCAAAACCCTATC
Human-PTK6-S2		TTTAGGGTTATGTGTTTTATAATT
Mouse-PTK6-F1	**Accession number :** NC_000068.8	TAGAGGGTGGATATTTGTTTAGGGA
Mouse-PTK6-R1		TTACATAAAATACCCCCAT
Mouse-PTK6-S1		TTTATAATTTTTAGTATTGGTTAAT
Mouse-PTK6-F2		GGGTAGTATGGGAGGTGAATT
Mouse-PTK6-R2		AGCTGGACATCACCTCCCACAACGAAAAAACCCACACCACCTCTA
Mouse-PTK6-S2		GTATGGGAGGTGAATTAA

**qPCR primer**			**Product length**
Human-PTK6-RTF	**Accession number :** NM_005975.4 NM_001256358.2	TGCCCCATTGGGATGACTG	221
Human-PTK6-RTR		GTACAGCGCCAGGATGTGTTT	
Mouse-PTK6-RTF	**Accession number :** NM_009184.2 NM_001356304.1	GGCTATGTGCCTCACAACTAC	76
Mouse-PTK6-RTR		GGGAGATGCAACCAAAGAAC	
Human and Mouse-GAPDH-RTF	**Accession number :** NM_002046.7 NM_001289726.1	CCCCTTCATTGACCTCAACTACAT	135
Human and Mouse-GAPDH-RTR		CGCTCCTGGAAGATGGTGA	

The original sequences detected:

Human-PTK6 S1:

CCGGACGGACGAGGAGCTGAGCTTCCGCGCGGGGGACGTCTTCCACGTGGCC

Human-PTK6 S2:

TACCTGGCCGAGAGGGAGACGGTGGAGTCGGAACCGTGCG

CTGGGTGTGGCTGCTTCCCGGCCTGTCATGAGGAAGTGGGACGGCCCGCCTGCC+1AC
GCCCAGCTCTGGGTGCTCCAGCTGGGCCACAGCCTGGTCCTGCCGCTGCGCCCGCCC
GCC**ATG**GTGTCCCGGGACCAGGCTCACCTGGGCCCCAAGTA*TGTGGGCCTCTGGGACT*
*TCAAGTCCCGGACGGACGAGGAGCT*GAGCTTCCGCGCGGGGGACGTCTTCCACGTGG
CCAGGAAGGAGGAGCAGTGGTGGTGGGCCACGCTGCTGGACGAGGCGGGTGGGGCC
GTGGCCCAGGGC*TATGTGCCCCACAACTACCTGGCCGAGAGGGAGACGGTGGAGTCG*
GAACCGTGCG

Human-PTK6 S1:

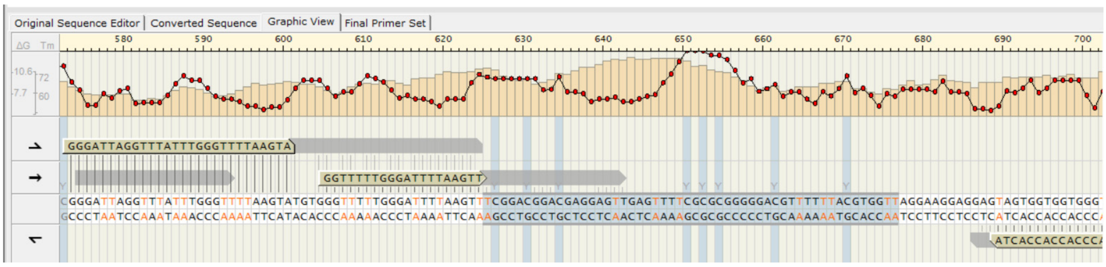

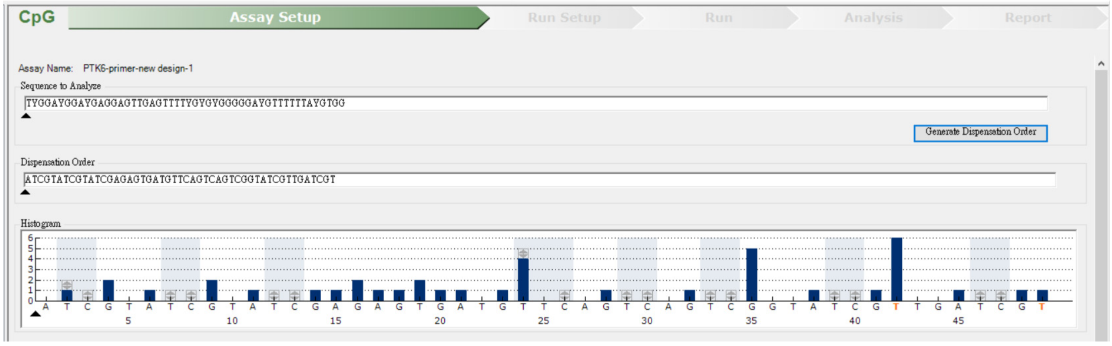

Human-PTK6 S2:

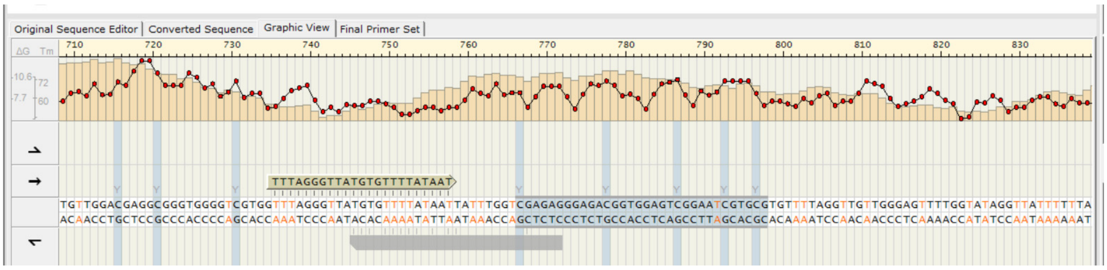

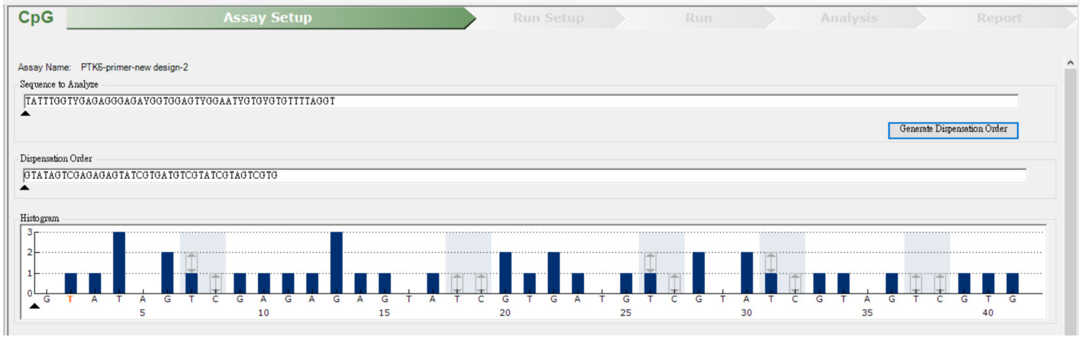

(Y means possible methylation sites; orange T means unmethylated cytosines were convert to thymine after bisulfite treatment and PCR)

Raw data of western blotting

Supplementary Fig. 2

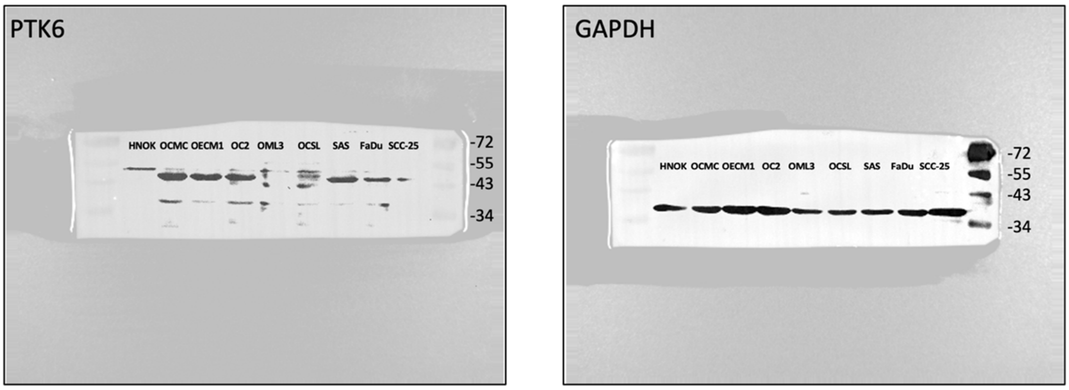

Figure 3a

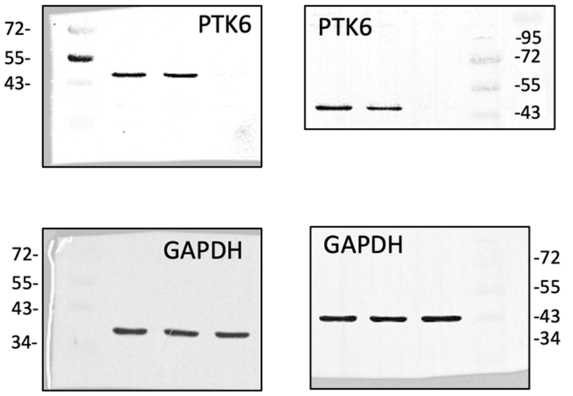

Figure 4a

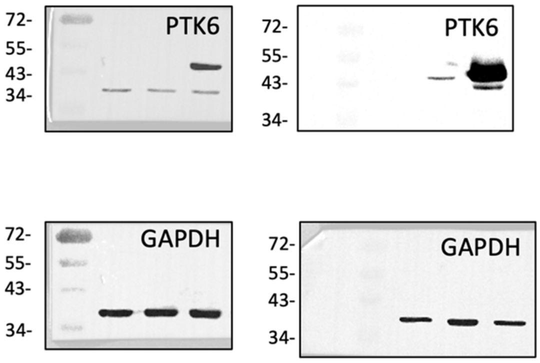

Figure 6

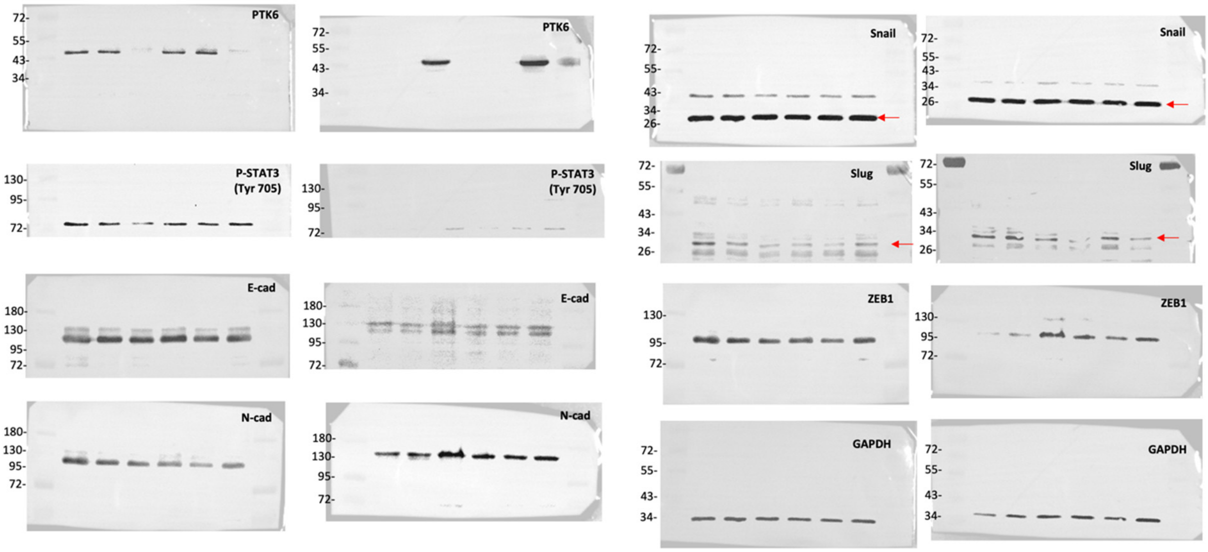

Supplement: Supplementary file 1 [file ijms-23-04495-s001.zip › ijms-1665679-SI.pdf]
